# Effectiveness of intraoperative intraocular lens use on improving surgical safety for dense cataract phacoemulsification: a randomized controlled trial

**DOI:** 10.1038/s41598-020-58597-0

**Published:** 2020-01-31

**Authors:** Mingbing Zeng, Rong Wang, Bing Cheng, Chengwu Yang, Yunxin Chen, Xialin Liu

**Affiliations:** 1 Zhongshan Ophthalmic Center, State Key Laboratory of Ophthalmology, Sun Yat-sen University, Guangzhou, 510060 P.R. China; 2 Hainan Eye Hospital and Key Laboratory of Ophthalmology, Zhongshan Ophthalmic Center, Sun Yat-sen University, Haikou, 570311 P.R. China; 3 Graduate center for Vision Research, SUNY College of Optometry, New York City, NY10036 USA; 4 Department of Epidemiology and Health Promotion, College of Dentistry, New York University, New York City, New York 10010 USA

**Keywords:** Lens diseases, Vision disorders

## Abstract

We designed this study to assess if surgical safety can be improved by intraoperative use of intraocular lens (IOL) for cataract phacoemulsification. We performed phacoemulsification cataract removal on 401 patients. We randomly assigned these patients into three groups: the standard setting (Group I, n = 134), with reduced vacuum and flow rate (Group II, n = 137), and with IOL insertion before the last quadrant was emulsified with standard setting (Group III, n = 130). The primary outcomes included the risk of posterior capsular rupture (PCR), ultrasound time, energy, and complications. The secondary outcomes included central corneal thickness (CCT), CCT changes, endothelial cells (ETC) counting, ETC loss, and the best corrected distance visual acuity (BCVA) measured on day 1, day 7 and day 30. If PCR occurred, we emulsified the residual lens materials after insertion of IOL and clean of the prolapsed vitreous. We found that the risk of PCR in Group III (0/130) was lower than Group I (9/134, corrected relative risk (RR) = 18.44, 95% CI: 1.08–313.56) and Group II (3/137, corrected RR = 6.64, 95% CI: 0.35–27.41). Group III showed better BCVA on day 1 and 7, less ECC loss on day 7 and 30, and less CCT increase on day 1 and 7. No cases converted to extracapsular cataract extraction. No residual lens materials misdirected into vitreous cavity. Intraoperative use of IOL can improve surgical safety for dense cataract phacoemulsification.

## Introduction

After decades of development, phacoemulsification for cataract extraction has become safer, faster, and more efficient than ever. It has become one of the most extensive and effective treatments for cataract surgeries. Developments of phacoemulsification technology include energy release model innovation, fluidics systems upgrading, and surgical techniques improvement^1^, among others. In particular, improved fluidics system has greatly enhanced the anterior chamber stability.

However, post-occlusion surge (POS) caused by anterior chamber instability is still an undesirable and inevitable consequence after occlusion break^2,3^. POS tends to occur in proportion to vacuum. For soft nucleus fragments emulsification, the phaco tip is often not totally occluded. Therefore, it can be emulsified with less energy and vacuum. After the core nucleus is emulsified, epinucleus is often *in situ*. This supports and protects the capsule from rupturing due to POS. But for dense nucleus, epinucleus rarely left after the nucleus was emulsified. Thus, the protective effect of epinucleus is lost. The last dense quadrant usually needs the highest preset energy and vacuum to be emulsified. This will inevitably cause POS. Without the protection of epinucleus, PCR occurs very often. Even worse, vitreous can lose or nuclear fragments can descend into the vitreous cavity. Emulsifying the last quadrant is still challenging and critical for complete removal of dense cataract, although there are some protective factors of POS and PCR. These factors include improved fluidics system, low-compliance tubing, aspiration bypass systems, and modified phaco needles^4^. In addition, how to deal with the residual nuclear fragments becomes a big problem if PCR occurs. The PCR can expand, and residual lens materials will descend into vitreous cavity if phacoemulsification is still being used. The conventional solution to this problem is converting to extracapsular cataract extraction (ECCE), which can cause lager astigmatism and corneal damage^5^. Dr. Om introduced the intraocular lens scaffold technique to prevent posterior capsule rupture in cases of Morgagnian cataract^6^. In our study, we use the similar skill. We evaluate surgical safety for dense cataract phacoemulsification and its effectiveness for preventing PCR or fall of the residual nuclear into vitreous cavity when PCR occurs.

## Results

Four hundred and one patients (401 eyes) met the inclusion criteria and enrolled in this study. We randomly assigned them into three groups. The distributions of age, gender, surgical eye were comparable among the three groups at baseline (Table 1). If both eyes of a participant underwent surgery, only the first qualified eye was chosen for comparison. Through simple randomization using random numbers method, we assigned 134 eyes to group I, 137 eyes to group II, and 130 eyes to group III. We classified nucleus hardness by the Emery-Little system. There were statistically significant differences in hardness among the three groups(p > 0.05). All patients showed up for the first two follow-up examinations. Five patients in the Standard setting group, four in reduced vacuum group and IOL pre-inserted group were absent to the last follow-up examinations. We excluded these patients from postoperative comparisons.

**Table 1:** Demographic data from the subjects in the three groups.

Demographics	Group I	Group II	GroupIII	P value
Patients/Eyes (n)	134/134	137/137	130/130	—
Mean age (y) ± SD	68.9 ± 10.1	69.0 ± 11.3	68.7 ± 10.5	0.472
Males/females (n)	65/69	64/73	63/67	0.671
Right/left eye (n)	66/68	67/70	65/65	0.601
**Nuclear Density**				
Grade IV (n/%)	84/62.7	80/58.4	78/60.0	0.509
Grade V (n/%)	50/37.3	57/41.6	52/40.0	0.701

ANOVA, otherwise the chi-squared test was used.

### PCR risk

The risk of PCR was quite different among the three groups. Nine cases in the Group I, three in group II, and zero in Group III had endured PCR. The rate of PCR occurrence was 6.72% in group I, 2.19% in group II, and 0.00% in group III. The risk of PCR in Group III (0/130) was lower than Group I (9/134, corrected relative risk (RR) = 18.44, 95% CI: 1.08–313.56), and lower than Group II (3/137, RR = 6.64, 95% CI: 0.35, 127.41).

Because extra maneuver can affect endothelial density and compromise the comparability among the groups, we excluded cases endured anterior vitrectomy from analysis, and compared for the rate of complications. If PCR occurred with remained lens fragments, we emulsified the nucleus after IOL and inserted them underneath the fragments. No case with residual nuclear fragments descended into vitreous cavity.

### Intraoperative outcomes

The mean and standard deviation of UST were 48.39 ± 16.7 seconds in group I,50.40 ± 20.3 seconds in group II, and 39.48 ± 11.3 seconds in group III (*p* < 0.01). The mean and standard deviation of CDE were 8.97 ± 4.6 in group I, and 9.08 ± 4.7 in group II, and 8.59 ± 3.50 in group III (*p* > 0.05).

### Best corrected visual acuity

On day 1 and day 7, the mean and standard deviation of BCVA and logMAR were 0.20 ± 0.11 and 0.00 ± 0.10 in group I, 0.19 ± 0.10 and 0.00 ± 0.09 in group II, and 0.17 ± 0.13 and −0.08 ± 0.05 in group III (p < 0.01). On day 30, the mean and standard deviation of BCVA were −0.10 ± 0.06 in group I, −0.10 ± 0.07 in group II, and −0.09 ± 0.07 in group III (*p* > 0.05).

### Central corneal thickness changes

**Figure 1:**
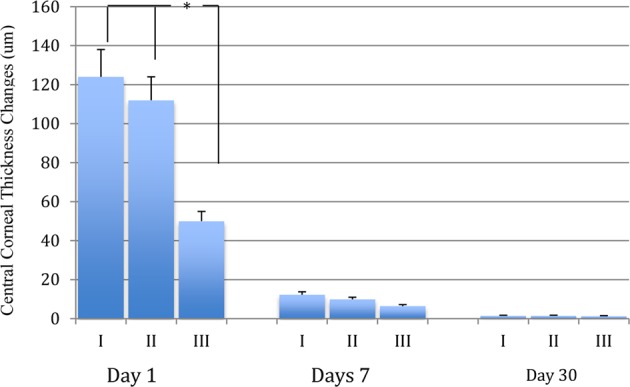
The postoperative corneal endothelial cells loss comparison on day 7 and 30. The central corneal endothelial loss difference between group III and II or I is statistically significant analyzed by ANOVA followed by LSD post-hoc multiple comparisons on day 7 and day 30. *P < 0.01.

**Figure 2:**
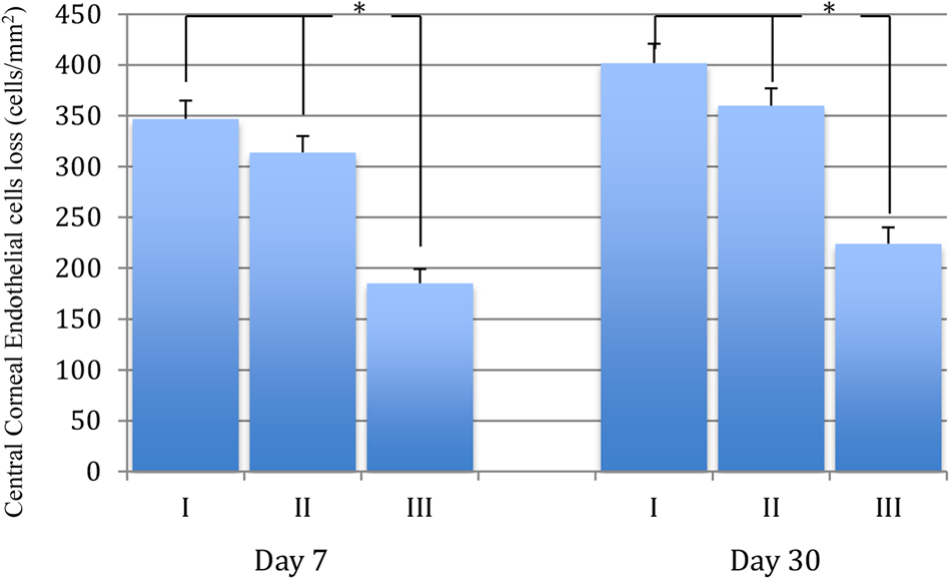
The postoperative central corneal thickness changes comparison on day 1, 7 and 30. The postoperative central corneal thickness changes difference between group III and II or I is statistically significant analyzed by ANOVA followed by LSD post-hoc multiple comparisons on day 1 and not significant oneday 7 and 30 visit. *P < 0.01.

On day 1 and day 7, the mean and standard deviation of CCT were 644.8 ± 47.1 um, 596.2 ± 20.6 um in group I, and 631.3 ± 39.9 um, 564.8 ± 12.4 um in group II, and 581.7 ± 11.4 um, 556.5 ± 10.3 um in group III (*p* < 0.01). On day 30, the mean CCT and standard deviation were 529.5 ± 11.8 in group I and 530.4 ± 12.1 in group II, and 528.4 ± 11.2 um in group III (*p* > 0.05, Table 2). On day 1 and day 7, the mean and standard deviation of CCT changes were 124.2 ± 14.1 um, 9.9 ± 1.1 um in group I, and 111.9 ± 11.6 um, 9.9 ± 1.1 um in group II, and 50.6 ± 5.7 um, 6.5 ± 0.9 um in group III (*p* < 0.01; Table 2, Fig. 1). On day 30, the mean and standard deviation of CCT were 529.5 ± 11.8 in group I,530.4 ± 12.1 in group II, and 528.4 ± 11.2 um in group III (*p* > 0.05, Table 2, Fig. 2).

**Table 2:** Postoperative central corneal thickness and its changes on day 1, 7 and 30.

	Groups	CCT	CCT Changes
1 day	Group I	644.8 ± 47.1	124.2 ± 14.1
	Group II	631.3 ± 39.9	111.9 ± 11.6
	Group III	581.7 ± 11.4	50.6 ± 5.7
	F	99.56	1900.80
	P	0.000*	0.000*
7 days	Group I	596.2 ± 20.6	12.4 ± 1.45
	Group II	564.8 ± 12.4	9.9 ± 1.1
	Group III	556.5 ± 10.3	6.5 ± 0.9
	F	84.748	920.00
	P	0.000*	0.000*
30 days	Group I	529.5 ± 11.8	1.46 ± 0.28
	Group II	530.4 ± 12.1	1.48 ± 0.29
	Group III	528.4 ± 11.2	1.32 ± 0.27
	F	0.608	0.363
	P	0.545	0.696

CCT: Central corneal thickness; CCT Changes: Preoperative CCT-Postoperative CCT.

*Statistical significance P < 0.01.

### Central endothelial cells loss

On day 7 and day 30, the mean and standard deviation of central corneal endothelial cell count were 2043 ± 188, 1938 ± 183 cells/mm^2^ in group I, 2100 ± 199, 1976 ± 185 cells/mm^2^, and 2260 ± 244, 2072 ± 151 cells/mm^2^ in group III (p < 0.001). On 7^th^ days after surgery, the mean and standard deviation of corneal endothelial cell loss in Group III was 184 cells (7.5%), compared to 350 cells (14.6%) in Group I and 312 cells (12.9%) in Group II (*p* < 0.05). The mean endothelial cell loss measured on postoperative day 30 was 401 cells (17.1%) in Group I, 360 cells (15.4%) in Group II, and 227 cells (9.8%) in Group III (*p* < 0.01, Table 3, Fig. 2).

**Table 3:** Endothelial cell and its loss comparison on day 7 and 30.

	Groups	ETC	ETCL
7 days	Group I	2043 ± 188	350 ± 17
	Group II	2100 ± 199	312 ± 16
	Group III	2260 ± 244	184 ± 15
	F	26.477	3984.11
	P	0.000*	0.000*
30 days	Group I	1938 ± 183	401 ± 18
	Group II	1976 ± 185	360 ± 18
	Group III	2072 ± 151	227 ± 15
	F	22.78	3813.04
	P	0.000*	0.000*

ETC: Endothelial cell; ETCL: Endothelial cell loss *: Statistical significance P < 0.01.

During the short follow-up period, we observed no postoperative complications in any patient, such as fibrin formation, synechiae, macrophages on the intraocular lens optic, or endophthalmitis.

## Discussion

PCR is the most common major intraoperative complication observed during cataract surgery. Literature report rupture rates varying from 0.5% to 10%^7^. If the PCR occurs, it is critical to remove vitreous and residual nuclear fragments from the wound, anterior chamber or vitreous cavity by anterior or pars plana vitrectomy. Failure to achieve this increases the risks of leakage, infection, or vitreous traction that may lead to cystoid macular edema or retinal detachment. Risk of PCR increases in difficult cases, including small pupil, pseudoexfoliation (PXF), intraoperative floppy iris syndrome (IFIS), and dense cataract such as mature, white, intumescent, or black cataract^8,9^.

PCR commonly occurs towards the end of the surgery when removing the last quadrant of the nucleus. The main reason is the posterior capsule is more exposed at the ending stages of operation. The improved fluidics system stabilizes the anterior chamber greatly. In addition, new generation machines can set vacuum at a level higher than ever before. When the tip of phaco needle is fully occluded by lens material, the whole of needle lumen, handpiece, and tubing connecting to the pump or cassette at a negative pressure (vacuum). This vacuum is set by the machine. When the lens fragment is emulsified or dislodged, the occlusion resolves suddenly. As a result, fluid rapidly flows from the small volume into the much larger volume across this pressure gradient. To maintain the anterior chamber (AC) volume, fluid must enter it through the irrigation line at the same rate. Otherwise, it will cause AC instability and cornea or posterior capsule collapse, which is called POS. For a given system, the amount of POS is directly proportional to the vacuum, inversely proportional to bottle height above the eye, and inversely proportional to tubing rigidity^10,11^. Besides surgical techniques, POS is the major risk of PCR, and is hard to avoid even for experienced surgeons.

For the soft-nucleus cataract emulsification, the preset highest energy and vacuum are often not essential to remove the last fragments. After the core nucleus is emulsified, epinucleus is often *in situ*. This is helpful to support and protect the capsule from collapsing. But for dense nucleus, such as white, black, mature, or morgagnian cataract, the epinucleus rarely remains. Higher vacuum and inspiration rate lead to higher amount of POS and higher risk of PCR. Therefore, dealing with the last chunk has become the major challenge of dense cataract emulsification. When POS occurs, the pre-inserted Sheets Glide separates the phaco tip from the posterior capsule, and prevents posterior capsule from contacting with phaco tip^12^. Reduced vacuum and aspirate rate along with increased bottle height may reduce the POS. But they can also reduce the surgical fluency. And they cannot completely prevent PCR. When managing the residual quadrants, we can use viscoelastic with high viscosity, and can inject it into the capsule. However, it is often aspirated before the nucleus emulsification and must be injected again. This increases the cost with extra viscoelastic. On the contrast, the inserted foldable IOL under the residual fragments has benefits. It plays a role similar to epinucleus, but without changing parameters, extra instrument, more viscoelastic, or surgical techniques. Plus, it can separate the phaco tip with the posterior capsule without worrying about PCR. Moreover, the reduced rate of PCR and higher success rate is helpful to enforce the surgeons’ confidence at dealing with difficult cases. Difficult cataract surgeries can greatly increase risks of PCR. These include prior history of pars plana vitrectomy, partial corneal opacity, etc. Previous vitrectomy and longer axial length with the zonular weakness can lead to deeper anterior chamber when phacoemulsification and make the operation more difficult. Eyes with corneal opacities or small pupil have increased risk of PCR as well, due to poor visualization of intraocular morphology and altered dynamics. Cases with detectable AC instability have dramatically increased risk of PCR, due to incision leakage, combined with POS. Inserted IOL is an essentially important method to prevent PCR when emulsifying the last nuclear fragments.

If PCR occurs, the viscoelastic was used to stabilize the chamber and support the remaining nuclear material. Hastily taking out the phaco tip will cause the anterior chamber depressurization and increasing the risk of PCR enlargement^13^. If the anterior vitreous membrane is not damaged, phacoemulsification can be continued. If the vitreous prolapses, the conventional way is to convert to ECCE. This is helpful for preventing the nuclear fragments from descending into the vitreous cavity. If the ECCE or no suturing manual cataract surgery to be converted, the corneal incision has to be enlarged or sutured. This results in increased astigmatism and corneal damage. If the phacoemulsification is continued to be used without further remedy, the residual nuclear fragments can descend into the vitreous cavity, and need pars plana vitrectomy to clear up. After implanting behind the lens fragments, the IOL is big enough to prevent the fragments from misdirecting.

The use of IOL as a scaffold is a known published technique for Morgagnian cataracts^6^. Large number of cases were necessary to carried out how much decreased Risk Factor benefit from this technique and what’s the influence to conventional surgical methods. In our study, nine cases in the Group I, 3 in group II and 0 in Group III had endured PCR. The rate of PCR is 6.72% in group I, 2.19% in group II and 0.00% in group III. The risk of PCR in Group III (0/130) was lower than Group I (9/134, corrected relative risk (RR) = 18.44, 95% CI: 1.08–313.56). The postoperative increased corneal edema, UCDA and the endothelial cells loss are benefit from the technique. For instance, the central endothelial cells loss was 17.1% in group 1 and it is 8.1% in group 3. In this study we also compared the conventional viscoelastic soft-shell technique with decreased vacuum and aspiration, he risks of PCR in Group III (0/130) was Group II (3/137, RR = 6.64, 95% CI: 0.35, 127.41).

The location of IOL implantation will depend on the tear size of PCR and whether or not the anterior capsule is intact. If the postcapsular tear is small, the IOL can be intracapsularly implanted after posterior capsulorhexis. If the PCR is not big enough to support IOL, the IOL has to be chosen zonular support. If PCR combined with large anterior capsule tear, the IOL has to be implanted into the anterior chamber. After the nuclear fragments were emulsified, the prolapsed vitreous body or residual cortex can be easily cleaned under the IOL by anterior vitrectomy. Then the IOL haptics can be fixed under iris, at ciliary sulcus or through sclera^14,15^.

When treating the last quadrant, the pre-inserted IOL is not only helpful for reducing the risk of PCR, but also decreasing the corneal endothelial damage from ultrasound energy. In our study, although the CDE difference was not statistically significant among the three groups, the increased central corneal thickness in the pre-inserted group was less than the other two groups 1 day and 7 days after operation. Also, the central corneal endothelial cell loss in the pre-inserted group was fewer than the other groups. A possible explanation could be the greater distance between the phaco tip and the cornea endothelium when treating the last quadrant of nucleus with a pre-inserted IOL. This is consistent with the results of other studies^16^. Because of the high risk of PCR caused by POS, the phaco tip was purposely put far from posterior capsule and closer to the corneal endothelium when emulsifying the last quadrant. On the contrary, with the protection of pre-inserted IOL, the phaco tip can be put far from corneal endothelium and the residual fragments can be emulsified quickly with less concern of PCR, in which situation the average UST was also found shorter than the other groups.

One of the difficulties in phacoemulsification is how to deal with the dense cataract, which is also one of the important aspects for residency training^17^. How to manage the last quadrant becomes the key in dealing with dense cataract. Effective use of IOL will improve surgical safety, shorten learning curve, and promote popularization of phacoemulsification for cataract extraction. For the cataract surgery, phacoemulsification mainly includes the following steps: making incision, capsulorhexis, nucleus emulsifying, cortex aspiration, and IOL implantation. However, the safety of the operation will be improved by inserting the IOL under the last quadrant at the end stage of phaco and may become one of the basic steps for successful dense cataract phacoemulsification removal.

In conclusion, IOL can be used not only as correction of aphakic eyes, but also as a tool to improve the surgical safety, especially for dense cataract phacoemulsification. The foldable IOL can play a role similar to epinulceus and be used as an ideal tool for reducing the risk of PCR when the last nuclear quadrant is emulsified. It can also be used for preventing residual lens fragments from descending into vitreous cavity and avoiding paras plana vitrectomy if PCR occurs.

## Materials and Methods

### Design

This is a randomized controlled clinical trial, implemented in an academic center of a university affiliated hospital. Through simple randomization, 401 patients were divided into three treatment groups at a 1:1:1 allocation ratio. Baseline demographics and major possible confounding factors were compared among the three groups, and then the main outcome measurements were compared. Specifically, Group III was the reference group, i.e., the comparisons was focused on Group III vs. Group II, and Group III vs. Group I. In order to avoid the potential bias brought by different surgeons, all of the surgeries were performed by the same experienced surgeon (Dr. Mingbing Zeng).

The study was carried out from Jan 01, 2015 to Dec 31, 2016 and approved by The Ethic Committee of Hainan Eye Hospital, Zhongshan Ophthalmic Center, Sun Yat-sen University. It was registered at Chinese Clinical Trail Registry on Sep 07, 2017 (http://www.chictr.org.cn/showproj.aspx?proj=21347) with the registration number: ChiCTR-INR-17012618. The study was informed by the CONSORT approach and the results were reported accordingly. The authors confirm that all ongoing and related trials for this intervention are registered.

### Participants

Four hundred and one eyes of 401 patients who have been taken phacoemulsification and IOL implantation were included in this randomized controlled study. The average age of the 201 women and 97 men was 65.4 ± 10.7 years (range 50 to 85).

Informed consent was obtained from all the patients before surgery, and the study was conducted under the principles of the Declaration of Helsinki. Institutional Review Board (IRB) and Ethics Committee approval was obtained prior to the onset of this study. The study was conducted at the Hainan Eye Hospital, National State Key Laboratory of Ophthalmology, Zhongshan Ophthalmic Center, Guangzhou, China, from January 2015 to December 2016.

Patients diagnosed as age-related cataracts only with nuclear hardness denser than Grade III according to the Emery-Little system, such as white, brown or black cataract, were recruited. Other inclusion criteria included a pupil diameter of 7 mm or larger; corneal endothelial cell count greater than 1200/mm^2^; and availability for regular follow up examinations. Patients were excluded if they had other vision-affecting or systemic disorders such as but not limited to, diabetic retinopathy, glaucoma, age-related macular degeneration, uveitis, or previous intraocular surgery.

### Study intervention

All of the surgeries were performed with a standard quick chop technique using Alcon Infiniti System (Alcon, Fort Worth, TX). The standard parameters were set with 100% fixed Torsional amplitude, 40% phaco power and the Intelligent Phacoemulsification (IP) on, 90% threshold vacuum limit, 100 ms phaco pulse on time, and 1.0 the ratio of longitudinal/torsional. The vacuum was 400 mmHg and the aspiration rate were 40 cc/min. For all groups, a MicroTip 0.9 mm ABS phaco tip (45°, Kelman) with Microsmooth High Infusion Sleeve was used. All patients received topical anesthesia of 0.5% proparacaine hydrochloride eye-drops (Alcaine) before surgery. A 3.0 mm wide self-sealing temporal clear corneal incision was made on the temporal side of the eye. ProVisc (1% sodium hyaluronate, Alcon) was used to reform and stabilize the surgical planes and protect the corneal endothelium. A 5.5–6.0 mm continuous curvilinear capsulorhexis was performed with a 26-gauge needle.

**Figure 3:**
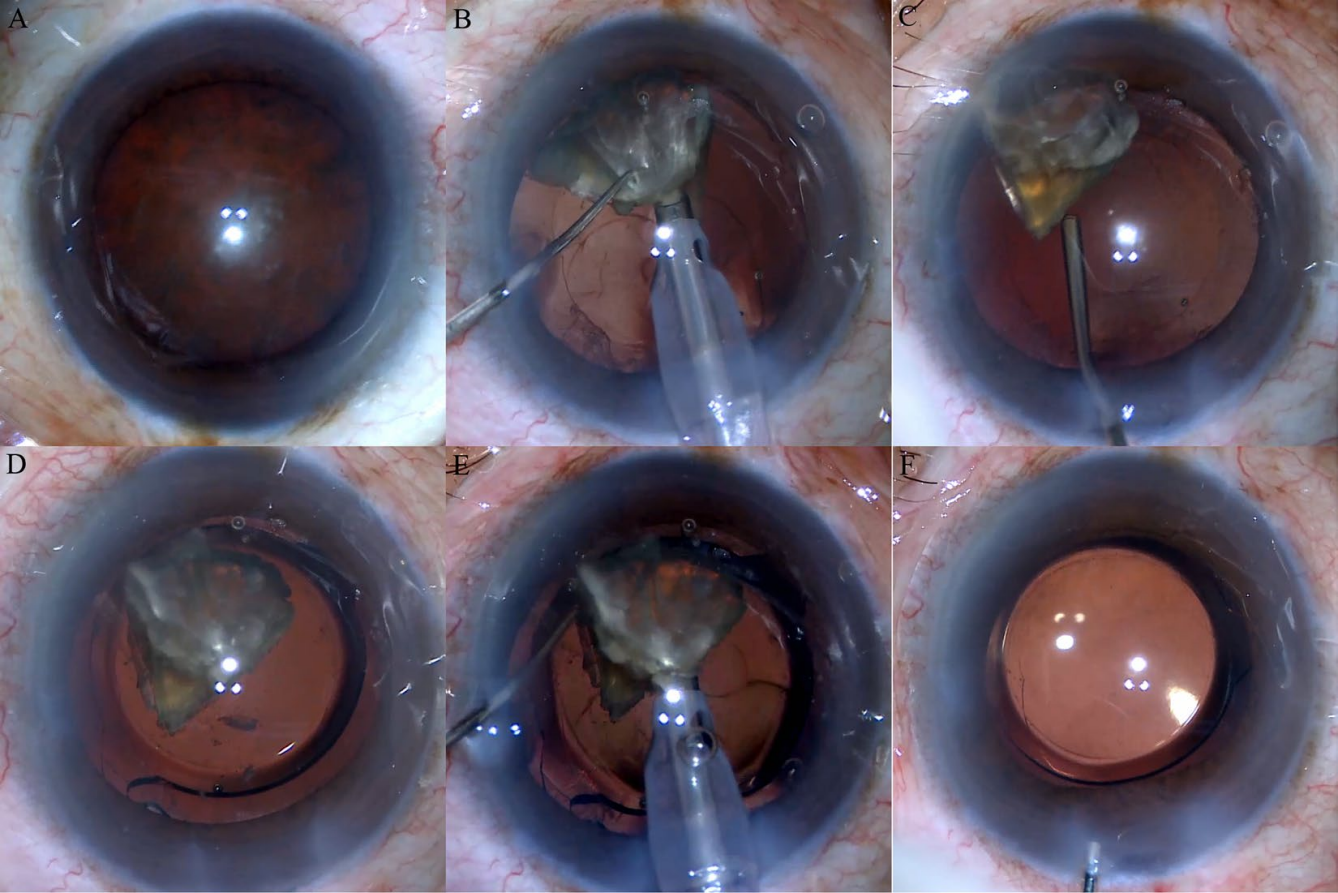
The intraoperative figures showing the last nuclear quadrant to be emulsified after IOL is implanted. (**A**) The dense cataract with nuclear hardness grade IV to V and little cortex; (**B**) The last quadrant to be emulsified after most of nuclear fragments were emulsified; (**C**) The remained nucleus is elevated outside the capsule with viscoelastic; (**D**) The IOL is inserted into the capsule and underneath the nucleus and then the nuclear fragment is pulled back into the center of anterior chamber. (**E,F**) The last nuclear quadrant is emulsified above the IOL.

After most of the divided fragments were emulsified, the last nuclear quadrant will be emulsified by different methods, by which all the eyes planned for surgery were then randomly assigned to one of the three groups. In the first group, the last quadrant was emulsified with standard setting as mentioned before. In the second group, under the fragments, the capsule was filled with ProVisc first and then the nuclear fragment was emulsified with reduced vacuum (250 mmHg) and aspiration rate (25 cc/min). In these two groups, the intraocular lenses were then inserted into capsular bag with the same injector system after the cortex was aspirated. In the third group, the ProVisc was injected into the capsule and push the last fragment into the anterior chamber and the foldable IOL (ZCB00, AMO) was inserted under the fragments, which were then emulsified with the standard setting (Fig. 3).

If PCR occurred without fragments left, anterior vitrectomy was performed to remove the prolapsed vitreous. If PCR occurred with the residual fragments, the phacoemulsification must be stopped or the PCR will be enlarged in no time and the fragments will easily descend into the vitreous cavity and have to be cleared by pars plana vitrectomy. The residual nuclear fragments were pushed anteriorly by viscoelastic and the IOLs were inserted underneath the fragments.If the posterior capsule tear is small and strong enough to support the IOL, the IOL can be implanted into the capsule. If the tear range is too large, the IOL has to be implanted into the ciliary sulcus. After the residual fragments were emulsified, the prolapsed vitreous was cleared by bimanual anterior vitrectomy.

### Main outcome measures

The used ultrasound time (UST), cumulative dissipated energy (CDE), PCR and other complications were recorded. The postoperative results were evaluated by another ophthalmologist, who was double-blind in the distribution of patients. The patients were followed up on the first day, the seventh day and the first month after operation. The best corrected visual acuity and complications after operation were recorded. The central corneal thickness was measured with the Anterior Segment Imaging VISANTE OCT 1000 (Carl Zeiss, Vertrieb Deutschland, Germany). The microscope (SP-2000 P, Topcon, Tokyo) and its ImageNet 2000, version 2.53 software (Topcon, Tokyo, Japan) were used to measure corneal endothelial cell counts. At least 100 cells in the center of each eye were selected to calculate the density of corneal endothelial cells. The average of the results of each three examinations represents the results of this patient. The statistical difference of UST, CDE, visual acuity, central corneal thickness and endothelial cell counts was evaluated by SPSS 22.0 for Windows 10.0 software (SPSS, Chicago, USA). The one-way ANOVA and LSD post-hoc multiple comparisons were selected to evaluate the difference between groups, and the bilateral P value was set at 0.05.

### Statistics and mathematics

At design stage, we anticipated that the PCR incidence rates in the three groups would be 6%, 2%, and 0% in Group I, II, and III. Using two-sided two-sample Fisher’s exact test with 80% statistical power at type I error level of 0.05, 131 participants per group are needed to detect the anticipated difference at PCR risk between Group III and I. However, 394 participants per group are needed in order to detect the anticipated difference at PCR risk between Group III and II. Given the resource constrains and in fact comparison between Group III and I is the primary research purpose of this study, we decided to recruit 131 participants for each of the three groups, i.e., 393 participants in total.

For the primary outcome, the PCR rate, Fisher’s exact test instead of ordinary Chi-square test was implemented, because we believe that there will be no PCR in Group III. For all of other outcomes, appropriate statistical analysis such as t-test, Chi-square test were used. The statistical significance level was set to the conventional level, i.e., 0.05.

## Data Availability

The datasets generated during and/or analyzed during the current study are available from the corresponding author on reasonable request.
